# Status of corneal endothelial cells in the presence of silicone oil in the anterior chamber

**DOI:** 10.1038/s41598-021-93338-x

**Published:** 2021-07-07

**Authors:** Machiko Shimmura-Tomita, Hiroko Takano, Yoshiaki Tanaka, Rina Takagi, Toshikatsu Kaburaki, Akihiro Kakehashi

**Affiliations:** Department of Ophthalmology, Saitama Medical Center, Jichi Medical University, 1-847, Amanuma-cho, Omiya, Saitama 330-8503 Japan

**Keywords:** Medical research, Outcomes research

## Abstract

To evaluate corneal endothelium damage with silicone oil (SO) presence in the anterior chamber after pars plana vitrectomy. We investigated the medical records of consecutive 54 eyes of 53 patients undergoing SO removal after pars plana vitrectomy with SO tamponade at Saitama Medical Center, Jichi Medical University, Japan. We recorded SO tamponade retention period, anterior chamber SO with gonioscope, area of SO attachment to the corneal endothelium before SO removal surgery, and the lens status. We then retrospectively investigated the correlation between SO presence in the anterior chamber and the decrease rate of corneal endothelial cell (CEC) density during SO tamponade. The average decrease rate of CEC density was 7.6 (0–38.1) %. The correlation between SO tamponade retention period and decrease rate of CEC density was high (*p* = 0.0001). However, there was no correlation between anterior chamber SO under gonioscope, SO attaching area, and lens status with the decrease rate of CEC density (*p* = 0.11, *p* = 0.93, *p* = 0.16). No correlation was observed between CEC loss and the existence of anterior chamber SO, although CEC decrease rate was relatively high after a long SO tamponade period. These findings suggest that SO presence in the anterior chamber may not directly injure CEC.

## Introduction

Silicone oil (SO) has been widely used as a tamponade for vitrectomy eyes since the 1970s^1–3^. SO is useful as a surgical material for vitrectomy, which conduces better therapeutic results for patients with proliferative vitreoretinopathy, proliferative diabetic retinopathy, recurrent retinal detachments, giant retinal tears, and traumatic retinal injuries^1,2,4–7^. However, SO has complications such as glaucoma, intraocular pressure elevation, corneal opacification, SO emulsification, and development of cataracts^8^.

There are many reports that show corneal endothelial cell (CEC) density decreases by SO tamponade^9–13^. It was reported that 12% of cases suffered corneal decompensation when SO tamponade period was extended over a long period, such as 12 months or longer^12^. Although the mechanisms involved in the effect of SO on CECs has yet to be confirmed, there are reports that absence of the iris-lens diaphragm is associated with decreased CEC density associated with SO tamponade^10,14,15^. Other reports show that CEC density decreased significantly in cases with large volumes of SO in the AC, and that CEC density in the superior half of the cornea decreased more due to the longer contact with SO compared to the lower half^16–18^. These reports suggest that CECs may be damaged by direct contact with SO. Nevertheless, many cases after SO tamponade do not suffer decreased CEC density in clinical practice.

On the other hand, corneal endothelium is damaged by surgical invasion alone. On average, endothelial cell density decreases by 10% one year after cataract surgery^19^. Pars plana vitrectomy and combination of cataract surgery with vitrectomy leads to modest postoperative decline in CEC density^20^. Intraocular surgery is considered to be more invasive to the corneal endothelium than extraocular surgery. Vitrectomy has been reported to decrease CEC density more than scleral buckling during surgery for retinal detachment^21^. In this study, we evaluated whether SO in the anterior chamber can damage the corneal endothelium after pars plana vitrectomy with SO tamponade.

## Results

### Patients’ demographics

Patients’ profiles are shown in Table 1. The mean age of patients was 60 (median 61, range 33–80) years old. There were 38 eyes of 37 male patients and 16 eyes of 16 female patients. The most frequent original disease for vitrectomy was complicated retinal detachment. The average number of intraocular surgeries prior to SO tamponade was 0.4 procedures. The most common simultaneous operation with SO tamponade was phacoemulsification and intraocular lens implantation in 31 eyes (57%). The lens statuses during SO tamponade was mainly pseudophakia (91%). The mean SO tamponade retention period was 328 ± 254 (median 275, range 63–1679) days. The mean follow-up period after SO removal was 1006 ± 914 (median 704, range 28–2320) days.

**Table 1:** Demographic data of the SO removal eyes.

Clinical characteristics		n (%)
**Age**	60 ± 12 (33–80)	
**Sex (Male : Female)**	38 : 16 eyes 37 : 16 patients	
**SO tamponade retention period**	328 ± 254 (63–1679) days	
**The average follow up period after SO removal**	1006 ± 914 (28–2320) days	
**Original disease**		
	Complicated retinal detachment	26 (48)
	Proliferative vitreoretinopathy	15 (28)
	Proliferative diabetic retinopathy	13 (24)
**Number of intraocular surgeries prior to SO tamponade **		
	0 procedures	36 (66)
	1 procedures	15 (28)
	2 procedures	2 (4)
	4 procedures	1 (2)
**Simultaneous operation with SO tamponade**		
	None	16 (30)
	PEA + IOL	31 (57)
	Pars plana lensectomy	4 (7)
	Segmental buckling	2 (4)
	SO removal	1 (2)
**Lens status when SO removal**		
	Pseudophakia	49 (91)
	Aphakia	4 (7)
	Phakia	1 (2)

SO, silicone oil; PEA, phacoemulsification; IOL, intraocular lens.

### Presence of SO in the anterior chamber

**Figure 1:**
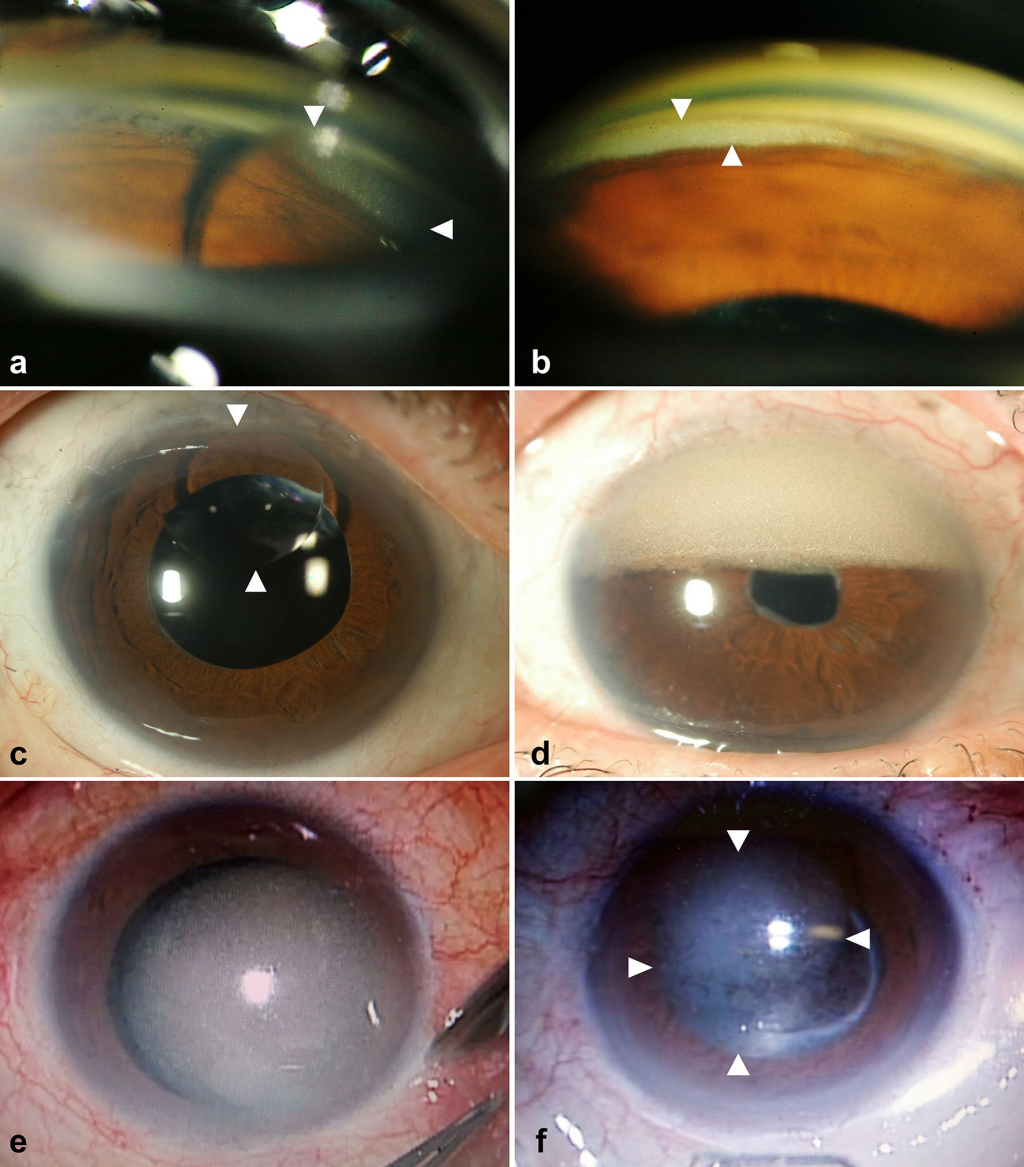
Anterior chamber silicone oil (SO) observation with gonioscope, slit lamp, and surgical microscope. Anterior chamber SO was observed with gonioscope in 19 eyes of 29 eyes. SO droplet was observed in the 1 o’clock angle direction by the gonioscope (**a**). A case in which fine particles and muddy SO are present in the upper angle only when seen with a gonioscope (**b**). Anterior chamber SO was observed with slit lamp in 13 eyes of 54 eyes. A case in which SO droplets are easily found in the anterior chamber by normal slit lamp microscopy examination (**c**). A case that seems to occupy approximately the upper half of the anterior chamber to the turbid fine granular SO (**d**). Anterior chamber SO was observed with surgical microscope at beginning of SO removal surgery in 33 eyes of 53 eyes. Because the patients are in supine position during surgery, SO present in the anterior chamber can be easily observed (**e**, **f**).

Anterior chamber SO was observed under gonioscope before SO removal in 18 eyes of 28 eyes (Fig. 1a,b), and the mean contact range of SO was 50 ± 18 (median 55, range 15–150) degrees of whole angle (360 degrees) (Table 2). Anterior chamber SO was observed with slit lamp before SO removal in 12 eyes of 54 eyes (Fig. 1c,d). SO was observed at beginning of SO removal surgery under the surgical microscope in 32 eyes of 52 eyes (Fig. 1e,f). The mean SO attaching area of these 32 eyes was 17 (median 14.1, range 0.2–68) percentage of total corneal endothelium. The lens statuses of the eyes examined SO attaching with surgical microscope were pseudophakia: 47 eyes (in the bag: 46 eyes, sulcus fixation: 1 eye), aphakia: 4 eyes, and phakia: 1 eye.

**Table 2:** Anterior chamber SO observation under various examinations.

Examination methods	Number of eyes
Under slit lamp	12 of 54
With gonioscope	18 of 28
With surgical microscope	32 of 52

SO, silicone oil.

### Comparison of corneal endothelial cell parameters before SO tamponade and before SO removal

**Figure 2:**
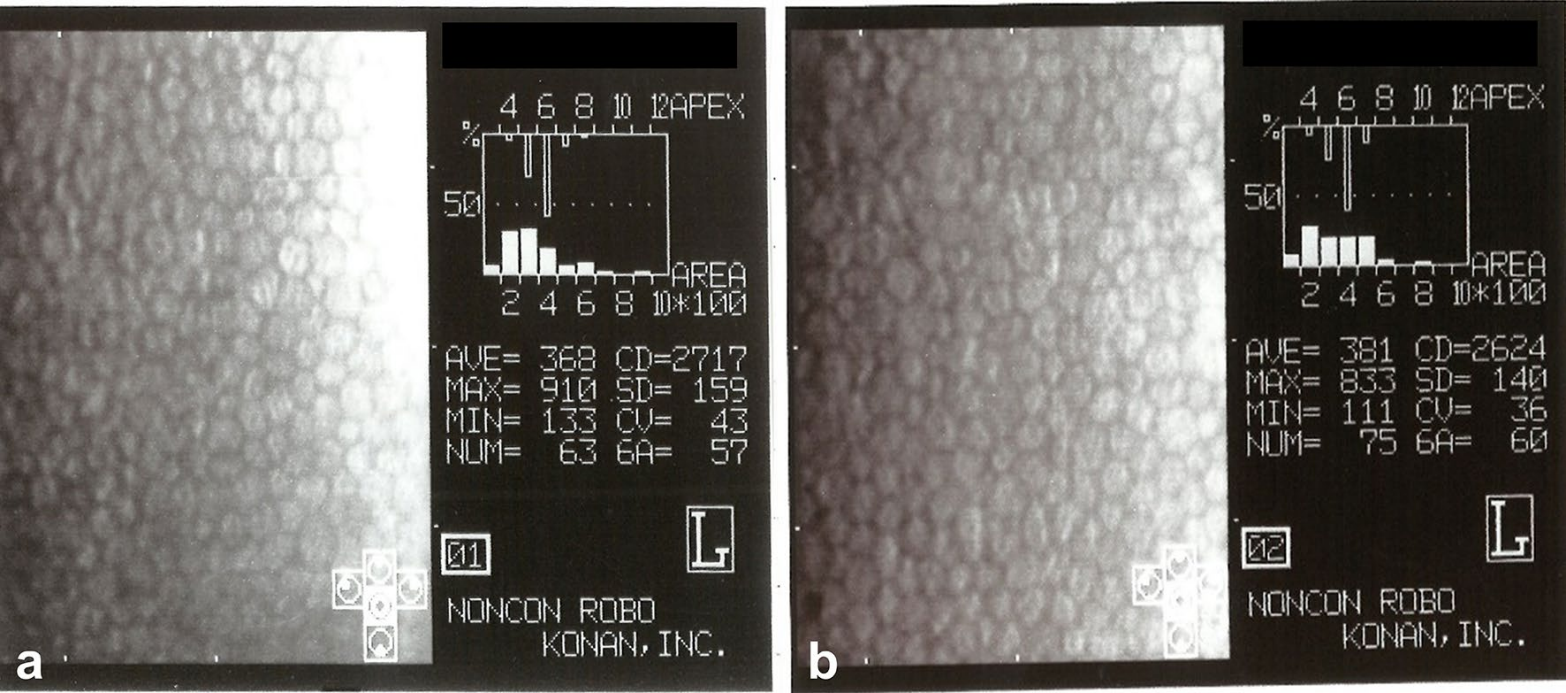
Representative specular microscopy findings before silicone oil (SO) implant (**a**) and before SO removal (**b**). This case was a pseudophakic 47-year-old man who underwent SO tamponade for retinal detachment recurrence. The period of SO tamponade was 258 days, and the period of SO in the anterior chamber was 189 days. During SO removal surgery, SO was found in the anterior chamber covering 14% of the corneal area. The decrease in endothelial cell density was 4.3%.

The mean decrease rate of CEC density between before SO implant and before SO removal was 7.6 (median 5.5, range 0–38.1) % (n = 55) (Table 3), and between before SO implant and last visit was 5.0 (median 4.4, range 0–11.8) % (n = 8). CEC density loss was observed in 38 eyes out of all 54 eyes (70%) (Fig. 2a,b). However, no CEC density loss was observed in 16 eyes (30%). 53 eyes had clear corneas, and only 1 eye had band keratopathy. There was no bullous keratopathy patient within the follow-up period. Due to CEC density decrease, the average endothelial cell area increased with SO tamponade (*p* = 0.018). The coefficient of variation of cell size and the percentage of hexagonal cells did not change with SO tamponade.

**Table 3:** Change of CEC between before SO implant and before SO removal.

	Before SO implant	Before SO removal	*P* value
	Mean ± SD (range)	Mean ± SD (range)	
CEC density (/mm^2^)	2566 ± 447 (1193–3508)	2447 ± 454 (1117–3125)	0.016
Endothelial cell area (μm^2^)	407 ± 104 (285–838)	433 ± 111 (320–895)	0.018
Coefficient of variation of cell size	36.3 ± 4.8 (28–49)	36.7 ± 5.0 (25–46)	0.65
Percentage of hexagonal cells	56.7 ± 8.2 (36–76)	57.1 ± 8.9 (34–77)	0.79

CEC, corneal endothelial cell; SO, silicone oil; SD, standard deviation.

### Correlation between corneal endothelial cell density reduction rate and various ocular parameters

**Figure 3:**
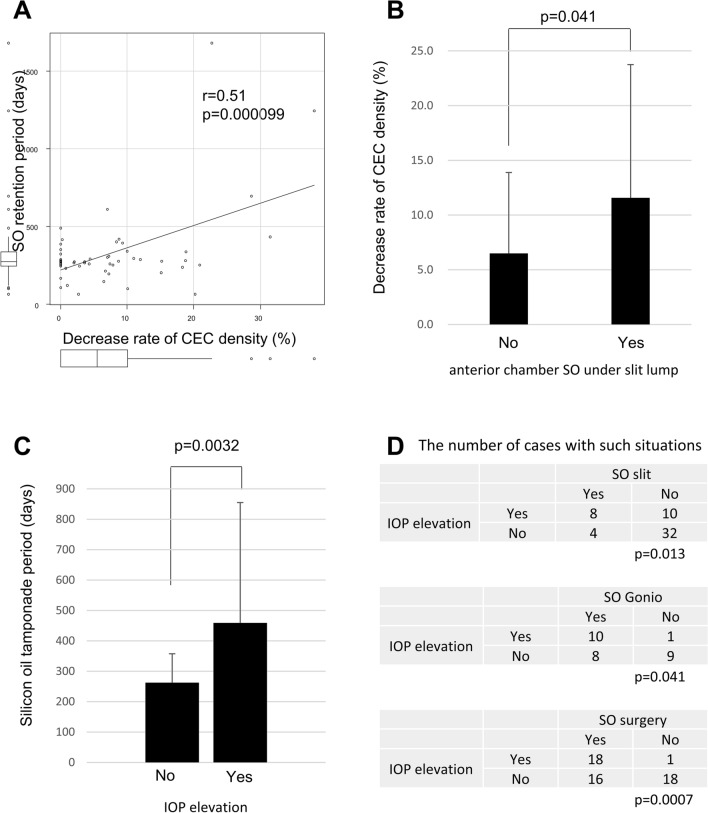
Decrease rate of corneal endothelial cell (CEC) density and intraocular pressure (IOP) elevation with some situations of silicone oil (SO) tamponade. High correlation was observed between decrease rate of CEC density and SO tamponade retention period (**A**). Correlation between anterior chamber SO under slit lamp and the decrease rate of CEC density (**B**). The SO tamponade period was significantly longer in cases with elevated IOP (**C**). Correlation between anterior chamber SO under gonioscope, anterior chamber SO under slit lamp, and SO attaching area with surgical microscope with IOP elevation (**D**).

The correlation between SO tamponade retention period and the decrease rate of CEC density was high (*p* = 0.0001) (Fig. 3A). On the other hand, there was no significant difference in the decrease rate of CEC density when comparing the SO retention period within 6 months and more than 6 months (t test, *p* = 0.25). In addition, there was correlation between anterior chamber SO under slit lamp and the decrease rate of CEC density (*p* = 0.042) (Fig. 3B). But there was no correlation between the number of previous eye surgeries, with or without simultaneous cataract surgeries during SO tamponade, anterior chamber SO under gonioscope, SO attaching area with surgical microscope, anterior chamber flare volume, sudden intraocular pressure elevation after SO tamponade, the highest intraocular pressure value during SO tamponade and lens status with the decrease rate of CEC density (*p* = 0.35, *p* = 0.17, *p* = 0.11, *p* = 0.93, *p* = 0.23, *p* = 0.38, *p* = 0.31, *p* = 0.16) . Furthermore, we investigated the relationship between groups with or without an intact posterior capsule and the decrease rate of CEC density, however, there was no statistical difference (*p* = 0.27).

### Correlation between intraocular pressure elevation and SO in the anterior chamber

In cases of elevated intraocular pressure, the SO tamponade period was significantly longer (*p* = 0.0032) (Fig. 3C). On the other hand, there was no significant difference in the incidence of elevated intraocular pressure when compared within 6 months and more than 6 months (Fisher’s extract test, *p* = 0.40). There was correlation between anterior chamber SO under gonioscope, anterior chamber SO under slit lamp, and SO attaching area with surgical microscope with intraocular pressure elevation (*p* = 0.04, *p* = 0.01, *p* = 0.0007) (Fig. 3D).

## Discussion

Decrease of CEC density after SO tamponade is one of well-known long term SO related complications^10–12^. However, is decrease rate of CEC density so high with SO tamponade? Boscia et al. reported that decrease rate of CEC density with SO tamponade was − 11.2% and minimally different from standard phacoemulsification with posterior chamber IOL implantation without trouble (− 8.3%) after 6 months^9^. Our result is − 7.6% decrease rate with SO tamponade after 10 months, then similar with standard cataract operations. Then we hypothesized SO might not harm CEC directly.

In this study, no correlation was observed between CEC loss and the existence of anterior chamber SO, although CEC decrease rate was relatively high in long SO tamponade period. These findings suggest that SO may not directly injure CEC. Several reports said that SO in the anterior chamber or SO in contact with corneal endothelium had the adverse effect on the corneal endothelial cells^16–18^. It was also reported that the intraocular fluid from SO tamponade patients contained higher concentrations of inflammatory cytokines when SO was present compared to after SO removal^23^. However, no correlation was observed between CEC loss and the existence of anterior chamber SO in our study. This finding suggests that SO may not directly injure CEC unlike the previous reports. On the other hand, as the duration of SO tamponade was longer, CEC density reduction was greater, so it seems likely that SO tamponade surgery itself may adversely affect CEC over a long period.

In this study, there was no difference in CEC density decrease rate between phakia or IOL with intact posterior capsule group, the eyes with iris-lens diaphragm and aphakia or IOL with posterior capsule rupture group, the eyes without iris-lens diaphragm. However, there are many reports that the absence of iris-lens diaphragm is associated with CEC density damage associated with SO tamponade. Goezinne et al. reported that an intact natural or artificial lens-iris diaphragm might provide a protective barrier against corneal endothelial cell damage from long term SO tamponade^14^. Cinar et al. reported that eyes that had undergone previous cataract surgery were more vulnerable to endothelial cell loss than phakic eyes after vitreoretinal surgery with SO tamponade^10^. Sugitani et al. reported that there was correlation between SO and CEC loss when AC barrier was compromised^15^. This point needs further study in the future.

According to the results of this study, the percentage of cases anterior chamber SO with gonioscope is higher than the proportion of cases anterior chamber SO under slit lamp. This is probably simply because SO can be observed under slit lamp if the amount of SO in the anterior chamber is large. However, it was an interesting result that the rate which SO was seen under the surgical microscope was higher than the rate under the gonioscope. This result suggests that even though the ophthalmologists consider that SO does not exist in the AC at the usual examination, SO may be in contact with the corneal endothelium every day when taking a supine position such as at bedtime in the patient's daily life. In fact, there were 2 eyes in which SO was not seen under a gonioscope, but SO was seen under a surgical microscope in this study. The small number of cases that have been examined about gonioscope is unfortunate point in this study, and further investigation is required in the future.

Iwata et al. reported that the decrease rate of CEC density was significantly higher in the aphakic eye than in the IOL eye when comparing SO tamponade period over 6 months’ cases, but the decrease rate of CEC was not different between IOL or aphakic eyes in SO tamponade period within 6 months’ cases^24^. In this study, the number of cases removed SO within 6 months was small (n = 7), comparison between cases in which SO was removed in less than 6 months after surgery and cases in which SO was removed in more than 6 months, there was no difference in CEC density decrease rate. In addition, there was no difference in the CEC decrease rate within 6 months and over 6 months depending on the lens status in this study (data not shown). However, the longer the SO tamponade period, the greater the decrease rate of CEC in our study, so this result supported that it is desirable to remove SO within 6 months as shown in the SO usage guidelines of the Japanese Retina and Vitreous Society^25^.

The intraocular pressure elevation cases were more increased as the SO tamponade period was longer in this study. Then it might be better to remove SO at a relatively early period to avoid intraocular pressure elevation. Furthermore, SO in the anterior chamber was apparently involved in the increase of intraocular pressure instead of the decrease of CEC density in this study. These results suggested that SO was more likely to affect the angle and the trabecular meshwork than the corneal endothelium. In fact, there are many papers describing SO and increased intraocular pressure. Over the half eyes (56.5%) after pars plana vitrectomy with SO tamponade in primary uncomplicated rhegmatogenous retinal detachments had an increase in intraocular pressure during the follow up period^5^. SO tamponade was a significant risk factor for elevated intraocular pressure (cumulative rates of elevated intraocular pressure was 28.4%) and about 65% of ocular hypertension patients who received SO tamponade had not stopped intraocular pressure-lowing drugs^26^. The mean intraocular pressure values in patients who had vitrectomy with SO tamponade a month after vitrectomy were significantly higher than the patients who underwent vitrectomy with air or saline solution tamponade^27^.

On the other hand, glaucoma patients were reported to have lower CEC density than patients without glaucoma of the same age group^28^. They hypothesized the mechanisms of that were direct damage from IOP, congenital alteration of the CEC in patients with glaucoma, glaucoma medication toxicity, or a combination of these. We may speculate that SO did not directly damage the endothelium, but rather caused the endothelium to be secondarily damaged by increasing intraocular pressure. But there was no correlation between IOP elevation and the decrease rate of CEC density in this study.

This study has limitations including a sample size, and a large variability between cases which may lead to unexpected bias. In addition, this study covers only one type of silicone oil (Silikon 1000®) since this product is the only one approved by FDA and Ministry of Health and Welfare of Japan. It should be noted that the results of this study are limited to this silicone oil.

In conclusion, no correlation was observed between CEC loss and the existence of anterior chamber SO, although CEC decrease rate was relatively high in long-term SO tamponade. These findings suggest that SO may not directly injure CEC.

## Methods

This retrospective study was approved by the Institutional Review Board of Saitama Medical Center, Jichi Medical University, Japan (S20-218) Due to the retrospective nature of the study, the requirement of an informed consent was waived. This study adhered to the tenets of the Helsinki declaration.

### Study subjects

Data from consecutive 54 eyes of 53 patients undergoing silicon oil (SO) removal after pars plana vitrectomy with SO tamponade at the Saitama Medical Center were included in this study. We excluded patients with the following abnormalities prior to SO tamponade: cases with corneal disease, cases with CEC density of 1000 or less, and cases using glaucoma eye drops. We also excluded cases in which gonioscopy or intraoperative findings were not available. We obtained all data from the medical records retrospectively.

We evaluated the change of corneal endothelial cell (CEC) between before SO implant and before SO removal by following parameters: CEC density, endothelial cell area, coefficient of variation of cell size, and percentage of hexagonal cells. We recorded SO tamponade retention period, anterior chamber SO with gonioscope, area of SO attachment to the corneal endothelium before SO removal surgery (the percentage of total cornea area), and the lens status. We retrospectively investigated the correlation between these findings and the decrease rate of CEC density.

Decrease rate of CEC density was calculated as the percentage of the difference between CEC density before SO implant and CEC density before SO removal divided by CEC density before SO implant.

The following preoperative patient characteristics were also collected: age, gender, the number of intraocular surgeries prior to SO tamponade, and original diseases.

### Surgical methods

All patients were operated by one experienced vitreoretinal surgeon (A.K.). Our vitrectomy technique with SO tamponade included the following: a standard 3-port 23-gauge vitrectomy with a trocar microcannula system. Vitreous removal was as complete as possible, with shaving of the vitreous base. The retina was mobilized by removing all epiretinal and subretinal membranes and strings, and performing relaxing retinectomies as a last resort, only in cases in which the retina remained rigid. A pars plana lensectomy without IOL implantation was carried out in eyes with anterior proliferative vitreoretinopathy (PVR) or rubeosis. Phacoemulsification and intraocular lens implant were carried out in eyes with cataracts. In all cases air-fluid exchange was performed and subsequently exchanged for SO (dimeticon 1000 centistrokes) (Silikon 1000®, Alcon, Fort Worth, USA). In some eyes with PVR, scleral buckling was performed. The decision whether to perform scleral buckling was made based on the amount of residual vitreous cortex and the number, size, and location of retinal break.

Indications for the removal of SO included attachment of the retina or complications such as ocular hypertension, epiretinal membrane, or severe after cataract. SO removal was performed by repeated fluid SO exchange to minimize the remnant of emulsified SO. Some additional procedures, such as endo-laser photocoagulation, intraocular lens implantation, removal of post capsule, removal of epiretinal membrane, removal of proliferative tissue, SF6 tamponade, or SO re-tamponade was carried out if they were needed during SO removal surgery.

Patient data, including demographic information, systemic and ocular parameters, clinical examination findings, and specular microscopy of the corneal endothelium using a noncontact specular microscope (Noncon Robo, SP 8000; Konan, Hyogo, Japan) were retrospectively collected from the patients’ medical records. SO attaching area with microscope was calculated assuming that the area of the entire cornea was 100% using the patients’ surgical video.

### Data analyses

Statistical analysis was performed with EZR (Saitama Medical Center, Jichi Medical University, Saitama, Japan), which is a graphical user interface for R (The R Foundation for Statistical Computing, Vienna, Austria, version 2.13.2)^22^. More precisely, it is a modified version of R commander (version 1.8-4) designed to add statistical functions frequently used in biostatistics. The changes in corneal endothelial cell parameters such as CEC density, endothelial cell area, coefficient of variation of cell size, and percentage of hexagonal cells between before SO implant and before SO removal were analyzed using the t-test. The correlation between decrease of CEC density and SO tamponade retention period, anterior chamber SO under gonioscope, anterior chamber SO under slit lamp, SO attaching area with surgical microscope, lens status, type of SO, anterior chamber flare volume, and the highest intraocular pressure value were analyzed using Pearson's product-moment correlation test. SO tamponade retention period, anterior chamber SO under gonioscope, anterior chamber SO under slit lamp, SO attaching area with surgical microscope, and lens status were compared between IOP elevation and no elevation groups using the Fisher exact test. The decrease rate of CEC density between posterior capsule intact group such as phakia or in-the-bag IOL eyes (n = 5) and not intact group such as aphakia or out-of-the-bag IOL eyes (n = 49), and between sudden intraocular pressure elevation after SO tamponade group (n = 22) and no elevation group (n = 32) were analyzed using the t-test. Probability (*p*) values of less than 0.05 were considered statistically significant.

### Ethics approval and consent to participate

All procedures performed in studies involving human participants were in accordance with the ethical standards of the institutional research committee and with the 1964 Helsinki declaration and its later amendments or comparable ethical standards. Informed consent was obtained from all individual participants included in the study.
